# The Impact of Backpack Loads on School Children: A Critical Narrative Review

**DOI:** 10.3390/ijerph15112529

**Published:** 2018-11-12

**Authors:** Michelle Perrone, Robin Orr, Wayne Hing, Nikki Milne, Rodney Pope

**Affiliations:** 1 Bond Institute of Health and Sport, Bond University, Gold Coast, QLD 4229, Australia; rorr@bond.edu.au (R.O.); whing@bond.edu.au (W.H.); nmilne@bond.edu.au (N.M.); 2 School of Community Health, Charles Sturt University, Albury-Wodonga, NSW 2640, Australia; rpope@csu.edu.au

**Keywords:** load carriage, children, school backpacks, configuration, impact

## Abstract

*Background*: Backpack loads of school students during school days have been suggested to range from 10% to as high as 25% of their body weight and may have a negative impact on their body. The aim of this review was to identify and review studies that have examined impacts of contemporary backpack loads on school children. *Methods*: A systematic search was conducted of the literature using key search terms. After relevant studies published in recent years were selected using strict inclusion and exclusion criteria, the studies were critically appraised and relevant data were extracted and tabulated prior to conducting a critical narrative synthesis of findings. *Results*: Twenty-one studies were included, ranging in methodological quality from poor to good (critical appraisal scores 22% to 77%). Students carried on average over 15% of their own body weight, which caused biomechanical and physiological adaptations that could increase musculoskeletal injury risk, fatigue, redness, swelling and discomfort. *Conclusion*: Considering the limited methodological quality and variations in foci across studies, further research is needed to elucidate: (1) the loads students carry around on a school day in their school backpacks and; (2) the biomechanical, physiological and physical effects of load carriage on students.

## 1. Introduction

A review by Mackenzie et al. [1] in 2003 of backpack loads carried by school students during a school day identified that children were carrying as much as 30% to 40% of their body weight. This review, while acknowledging that no critical maximal load had been established (to address back pain), recommended around 10% of the child’s bodyweight as a maximum limit. The following year, a review by Brackley and Stevenson [2] stated that the majority of work considered the loads carried by children to be above recommended limits, likewise recommending a maximal load of between 10 to 15% of the child’s bodyweight. Since these reviews, more recent research has suggested that these loads are lighter and in some instances may be meeting this recommendation, with loads ranging from 10% [3,4,5,6] to 25% [7,8,9,10,11] of the school child’s bodyweight. However, this recent research, in agreement with the earlier reviews, also suggests that these loads have a negative impact (e.g., increased forward lean, pain, skin pressure) on children’s bodies [3,4,5,6,7,8,9,10,11].

Items carried by students in their day-to-day school bags have been found to include, but are not limited to, laptops, books, pencil cases, scientific calculators, sports uniforms, school day uniforms and sport-specific training clothing, up to three types of shoes (sport-specific footwear, school leather shoes, and general runners), lunch boxes and full water bottles [12]. To address the requirement to carry backpacks weighted down by all these items, the review by Mackenzie et al. [1] recommended the provision of school lockers. However, with school lockers being removed from many schools in recent years due to vandalism and security fears [13], anecdotal evidence suggests that school children are often required to carry their backpacks, loaded with all of these items for long periods of time. In addition, loads may be increasing due to increases in the size of school backpacks and increases in loads carried due to changes in the school curriculum (e.g., more homework being assigned, and increases in after-school/extracurricular activities) and the carriage of emerging items (like laptops) [14,15,16]. The need for children to have to carry a full day’s class schedule of schoolbooks, in addition to other items and supplies (e.g., sporting and musical equipment for after-school/extracurricular activities), throughout the day is thus a growing concern [15]. The sight of young children marching off to school heavily burdened by backpacks, coupled with a rising rate of non-specific back pain among schoolchildren, has led to increasing parental and community unease [15].

Given the loads being carried by children, whose musculoskeletal systems are still developing [12] and are undergoing rapid physical development [17], it is not surprising that this load carriage has been associated with musculoskeletal injury concerns [18]. Concerns associated with load carriage are not only prevalent in school children but in workplace occupations as well. Tactical populations, like those serving in law enforcement, firefighting, first response and the military, by nature of their occupations, are also required to wear and carry loads that can range from 8 kg in law enforcement officers [19] to 45 kg or more in army personnel [19]. In these well-trained adult populations, injuries like rucksack palsy (also identified in school children who carry loads [2]) and in the lower back are associated with load carriage [20]. With the growing spine vulnerable to physical stresses, and physical stress being a predisposing factor in adolescent spinal pain [17], the supposition that the carrying of a backpack is a contributing risk factor to adolescent low back pain bears merit [15,21]. This supposition is supported by Sheir-Neiss et al. [22], who found that backpack use and backpack weights were independently associated with back pain in adolescent school children. With research in young military recruits suggesting that once someone is injured carrying a load they are more likely to be injured again [23], any injuries induced by carrying a school backpack should be concerning for all involved and justify the concerns raised by parents, educators and health care professionals regarding the role of heavy backpacks [1,21].

In essence, as their school loads increase, the bodies’ of students may be subjected to the same stresses as those faced by trained tactical professionals. However, unlike trained tactical professionals, the school student is required to carry their loads almost daily for a period of up to 12 years, without the benefit of specific physical training to endure such stress and prior to reaching full maturity of their musculoskeletal system. Furthermore, with the levels of obesity in school children rising [24] and the overall relative demands placed on a school child’s body to carry these loads being greater in sedentary and overweight children [1], the potential impacts of schoolbag loads on the child may be increasing. Therefore, the aim of this systematic review was to determine the impacts of contemporary backpack loads on school children through a critical appraisal and synthesis of key findings from recent studies. The findings from this research will inform future research on this topic and assist with the development of risk management strategies for school backpack loads.

## 2. Materials and Methods

### 2.1. Search Strategy

To identify and obtain relevant original research for this review, key literature databases were systematically searched using specific keywords relevant to the topic. The selection of keywords was guided by a review of terms used in related relevant published articles. To improve the relevance of search results, filters that reflected study eligibility criteria were applied to each database. The databases were searched for specific key words (see Table 1).

### 2.2. Screening and Selection

Articles identified from the database search were first screened by title and abstract to identify and remove duplicates and articles that did not meet the review’s inclusion criteria (see Table 1). Publications reporting studies that used the same data set, analysed in similar fashion, as another study were treated as duplicates and were removed, along with duplicates arising from searching two different databases. All remaining articles were then obtained in full text and subjected to the review’s inclusion and exclusion criteria to determine their eligibility for inclusion in the review, identifying a final set of eligible articles for inclusion.

### 2.3. Inclusion and Exclusion Criteria

Inclusion criteria for this review were as follows: (a) human subjects, (b) English language, (c) children 6–18 years, and (d) publication date within the preceding 15-year period. Subject matter experts were consulted to decide upon the 15-year date range employed for this review. Reasons for this time period included the emergence of the use of the traditional (non-touch screen) notebook computers in the classroom and the use of personal laptop computers, which saw a dedicated surge around 2004 [16], with US school districts subsequently encouraged to provide students with personal laptops in 2010 [25].

Exclusion criteria were as follows: (a) studies that addressed backpack carriage in children with disease and illness; (b) studies of backpack usage with non-school children; (c) studies on backpack usage but with no relevance to school children (e.g., backpack-back pain complexity and the need for multifactorial safe weight recommendations [26]); (d) systematic reviews on backpack usage; and (e) articles that were not reports of original research (e.g., policies or guidelines on how to wear a backpack correctly).

### 2.4. Critical Appraisal and Data Extraction

Following study selection, the included studies were critically appraised using the Downs and Black checklist [27]. The checklist has 27 items designed to assess the methodological quality of randomized controlled trials and non-randomized studies and identify the strengths and weaknesses of these studies, and has been used in previous reviews [27]. Most of the items are scored on a dichotomous scale, awarding one point for a ‘yes’ answer and zero points for a ‘no’ answer or where the answer cannot be determined from the published report of a study. Item 5 on the checklist, however, is scored on a three-point scale, awarding two points for ‘yes’, one point for ‘partially’, or zero points for ‘no’ or ‘unable to determine’. The final question in this checklist, which assesses the statistical power of the study, is normally scored on a scale of 0–5 based on the study’s sample size relative to requirements to ensure adequate power. This question was modified for the current review to give one point for a ‘yes’ answer, indicating the authors of the study reported a power analysis, or zero points for a ‘no’ answer, indicating the authors did not report a power analysis. This modified approach to the checklist has been used previously to limit subjectivity in scoring [28]. Through this modified approach, the maximum possible raw score became 28, as opposed to the original maximum score of 32, and the raw score for each included study was converted to a percentage score.

All papers were independently scored by two reviewers (Michelle Perrone and Robin Orr), with the level of interrater agreement determined via a Cohen’s Kappa analysis. Final scores were determined by consensus between the two raters. Where agreement was not achieved through consensus, a third reviewer (Rodney Pope) determined the final score.

Kennelly’s [29] grading system awards a rating based on the Downs and Black raw score given by the raters. However, as Question 27 was modified for this review, the Kennelly grades were modified and presented as percentages to accommodate the modified Downs and Black checklist total raw score (now out of 28 and subsequently converted to a percentage score). On this basis, the grading system applied was as follows: >61% was graded as ‘good’ quality, 45–61% as ‘fair’ quality, and <45% as ‘poor’ quality. This modification has been previously used in critical reviews [30].

Once the critical appraisal of the studies was completed, pertinent data were extracted from the included studies and tabulated. Information extracted from the studies provided a concise and systematic overview of key attributes of, and findings from, included studies. The key table headings were: (a) lead author, title and publication date, (b) demographics of the participants, (c) load conditions, (d) outcomes measures used, (e) main findings and (f) the critical appraisal of studies (CAS), expressed as a percentage. A critical narrative synthesis of findings from the included studies was then conducted.

## 3. Results

**Figure 1:**
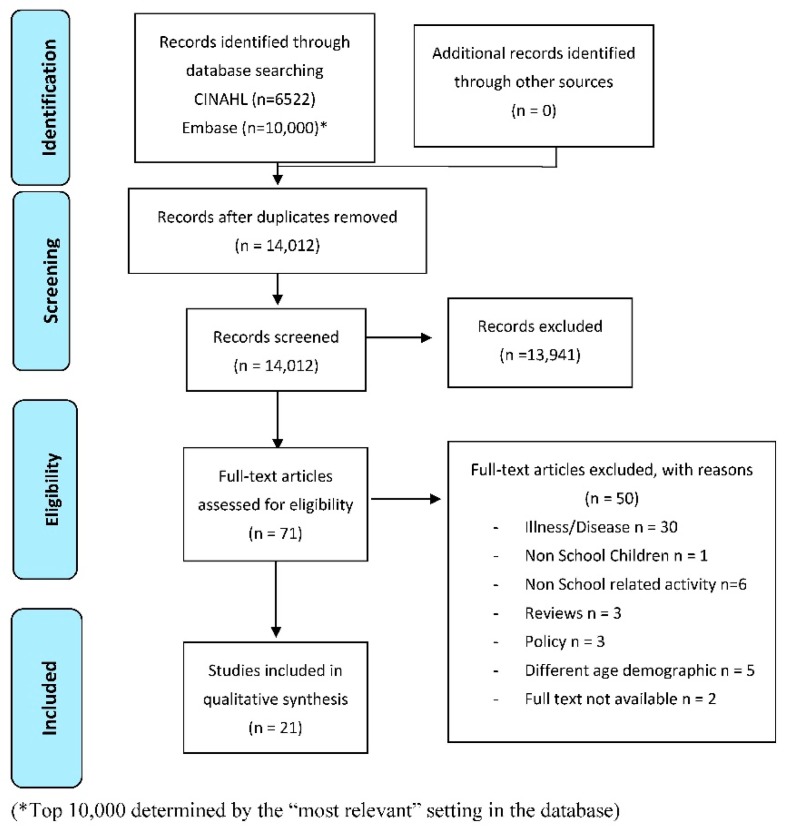
Preferred reporting items for systematic reviews and meta-analyses (PRISMA) diagram [31] detailing results of the search, screening and selection processes.

The results of the search, screening and selection processes are shown in Figure 1 with key outcomes extracted and compiled in Table 2.

### 3.1. Critical Appraisal of Studies (CAS)

The final CAS percentage scores, indicating the methodological quality of each study, are presented in Table 2, along with information regarding the outcome measures that were used in each study and the study’s findings. The Cohen’s kappa analysis revealed an interrater agreement level (k = 0.728) indicating ‘substantial agreement’, as per Viera and Garrett’s interpretation [40]. Fourteen studies were graded as ‘good’ quality studies [3,4,5,6,7,9,10,11,13,14,15,18,38,39], six were graded as ‘fair’ quality [8,32,33,34,35,37], and one was graded as ‘poor’ quality [30]. The mean CAS percentage for methodological quality of the included studies was 59%, (‘fair’ quality), with a high score of 77% (‘good’ quality) [13,15,18] and a low score of 22% (‘poor’ quality) [36].

### 3.2. Participant Characteristics

Of the 21 studies eligible for review (Table 2), five were conducted in China [8,9,11,32,33], three were conducted in each of the USA [13,15,39] and Italy [6,18,35], two conducted in Spain [35,38], two in India [10,36], two in Poland [4,5], and one study was conducted in each of the following countries: Ireland [7], Turkey [34], South Africa [14] and France [3]. Five of the studies involved only male participants [8,9,10,11,34], one study involved only female participants [34], 13 studies involved both male and female participants [3,4,5,6,7,13,15,18,32,35,37,38,39], and three studies did not specify the gender of participants [14,36,41]. In regard to ages of the students, 12 studies focused on primary school children aged 6–13 years [3,4,5,6,7,8,9,11,18,32,34,38], six focused on middle school children aged 13–15 years [10,13,15,37,38,39], two focused on secondary school children aged 13–17 years [35,37], and one study focused on both primary (10–12 years) and secondary (12–14 years) students [33]. The participant sample sizes of the studies varied. Two studies involved large samples encompassing 3852 [37] and 1403 [35] students, eight studies had sample sizes that ranged from 162 to 529 [4,5,6,7,10,14,15,18] students, six studies had sample sizes that ranged from 20 to 41 students [3,33,39] [34,36,38], and four studies had relatively small samples that ranged from 4 to 15 [8,9,11,32] students. One study did not mention its sample size [13].

### 3.3. Nature of the Load Carriage

When considering the nature of the loads being carried, all but four studies [9,32,33,40] involved students utilizing their own school bags, whereas the four [9,32,33] divergent studies used dummy/commercial/mono backpacks (Table 2). Four studies observed students carrying backpacks around the school on a daily basis [3,8,9,35], two studies stated students used lockers at school or left bags next to their classroom desk [5,15], one study stated students only carried backpacks over a short distance [7], and five studies did not state the nature of the backpack carriage at school [4,6,18,32,33]. It is important to state that none of the included studies investigated and itemized the contents of the loads carried by students in the real world (i.e., school environment). This lack of itemization of loads is a gap that demands further research, especially given technological advances and the fact that the school students’ backpack loads are evolving as a result.

### 3.4. Contexts of Measurement

Fourteen studies collected data in a controlled environment (Table 2) (e.g., laboratories and rooms) [4,6,7,8,9,10,18,32,33,34,35,36,38,39], five through visual observation in a controlled environment and questionnaires [5,13,14,15,37], and two tested their participants on treadmills [3,11]. Seven of the 21 studies reported set distances and times over which measurements were taken in the testing conditions (e.g., three minutes, 20 min, eight meters, 10 m, 400 m) [8,9,10,11,18,33,39]. Fourteen studies reported that testing was conducted with students standing still [4,5,6,7,10,13,14,15,32,34,35,36,37,38] for periods of 20 s [8] to 3 min [32]. No studies were conducted outside in the school yard and no studies calculated distances that loads were carried between homes and the school, suggesting that further research is needed to address these deficits identified previous research.

### 3.5. Load Weight

Ten studies (Table 2) weighed students and their backpacks [5,6,8,10,11,14,18,35,37,38], and three reported weighing the backpack only [9,13,35]. In these 13 studies, the mean backpack loads ranged from 5.5 kg [5] to 7 kg [5]. Four studies involved the testing of backpack load carriage using backpacks weighing 10% of the student’s body weight (BW) [3,4,5,6], five reported testing of backpack load carriage at 15% of the student’s body weight [18,32,33,34,38] and five reported the testing of backpack load carriage at 10%, 15%, 20% and 25% of the student’s body weight [7,8,9,10,11].

### 3.6. Biomechanical Outcome Measures

A variety of biomechanical impacts of load carriage were investigated (Table 2). These biomechanical impacts of load carriage included changes in postural parameters and changes to gait parameters.

#### 3.6.1. Posture-Related Biomechanical Measures

The impacts of backpack load on posture focused primarily on changes in postural angles. Drzal-Grabiec et al. [4] examined postural parameters, including habitual posture, with the backpack on the participant’s left and right shoulders. The student participants, in this study, exhibited a significant reduction of thoracic kyphosis during load carriage when compared to no load being carried (*p* < 0.05). In particular, the angle of thoracic kyphosis increased between measurements (*p* < 0.05) [4]. Similarly, Ramprasad et al. [10], who also examined changes in various postural angles with different backpack weights, found that the craniovertebral (CV) angle changed significantly after 15% BW was reached in backpack loads (*p* < 0.05). Head on neck (HON) and head and neck (as a single unit) on trunk (HNOT) CV angles were found to significantly change after a load of 10% of BW was reached (*p* < 0.05) [10]. However, trunk and lower limb angles, which also altered significantly, changed after only a load of 5% of BW was reached in the backpack (*p* < 0.05) [10]. Furthermore, in a study by Chow et al. [32] changes in spinal curvature and repositioning of the student’s centre of gravity (CG) were observed, when backpack loads exceeded 15% of the student’s body weight, at different CG locations (anterior and posterior at T7, T12, L3) [32]. This is the same percentage of body weight found to induce biomechanical changes that were reported by Ramprasad et al. [10].

Pau et al. [6] assessed the effects of backpack load carriage on plantar pressure distribution and spatio-temporal gait parameters among children. Measures were obtained during both quiet standing and walking, both with and without the backpack in 208 participants. More than half of the children in this study exhibited a backpack/body mass ratio higher than 15%. Pau et al. [6] found that backpacks introduced significant increases in overall center of gravity (CG) contact area (up to 10%). They also observed that plantar pressure peaked in the forefoot and midfoot regions when loads were carried by children, with a significant shift in the average position of the center of pressure towards the forefoot and midfoot, indicating the body’s attempt to restore the initial balance conditions challenged by the load. Pau et al. [6] suggests that heavy loads and significant exposure may increase the risk of foot discomfort and onset of foot structure alterations or pathologies.

#### 3.6.2. Gait-Related Biomechanical Measures

Six studies [3,6,9,11,37,39] investigated the impact of backpack loads on parameters of gait. Three of the studies [9,39], compared both unilateral (single shoulder backpack carry) and bilateral (two-shoulder backpack carry) loads, whereas one study [37] looked at a unilateral load only, comparing a loaded side to an unloaded side. In the study by Hong et al. [9], the researchers examined the effects of carrying methods (backpacks and single-strap athletic bags) and loads on phases of gait and ground reaction forces during stair ascent and descent in children. The threshold load that caused a significant increase in the peak forces observed, regardless of bag type or stair mode, was 15% of body weight [9]. Hong et al. [11] demonstrated that stride length decreased in the right and left legs when the backpack was worn over one shoulder but increased in both legs when the backpack was worn over both shoulders.

Contrary to the findings above, Cottalorda, et al. [3] found that when children carried a backpack (on one or two shoulders) they walked with longer stride, stance and double stance than when walking without a backpack. Connolly et al. [39] found an increase in double limb support when weight was added to the backpack as a counter measure to causing increased instability during gait. They also suggested that when carrying the backpack over one shoulder, the individual may lean in a direction opposite to the force (or load). As such it is not surprising that Pau et al. [6], while not directly observing spatio-temporal gait parameters, observed a significant increase (up to 25%) in plantar pressure, especially in the forefoot.

The consequences of the aforementioned changes in gait parameters from increased load, such as reduced stride length might be explained by the findings of Ozgul et al. [34]. Ozgual et al. [34] found peak ankle dorsiflexion and hip extension, as well as range of pelvic rotation, all decreased in adolescents carrying loads. Furthermore, knee flexion, a loading response for the support phase of gait, increased on the loaded side relative to the unloaded side when loaded walking was compared to unloaded walking. Decreased maximum hip extension during late stance, increased hip adduction, an elevated pelvis and increased anterior pelvic tilt were seen on the loaded side, and if the pelvis was lowered, ankle dorsi flexion increased and the hip was abducted on the unloaded side as a counter effect, when bags were carried on one side [34]. Thus, Ozgul et al. [34] suggest that both unloaded and loaded sides were affected by asymmetrical backpack carriage, putting more load on the lumbar vertebral joints, altering frontal knee biomechanics and contributing to lower back pain. These findings however do require further research given the ‘fair’ methodological quality (CAS score of 59%) of this research.

### 3.7. Physiological Outcome Measures

Vieira et al. [38] examined the influence of backpack type on lung function and respiratory muscle strength (Table 2) in both primary and secondary school children carrying a backpack under three controlled conditions: (1) with bilateral shoulder straps used to carry the load evenly on both shoulders, and carrying 15% of the student’s body weight; (2) with bilateral shoulder straps used to carry the load over one shoulder, and carrying 15% of the student’s body weight; and (3) with padded mono adjustable shoulder straps, carrying the load over one shoulder and across the body, and carrying 15% of the student’s body weight. The observed restrictive effect of load carriage and the resulting decrease in expiratory muscle strength were more pronounced for the backpack with a mono shoulder strap, suggesting a double strap backpack is preferable [38].

### 3.8. Measures of Physical Discomfort and Pain

Seven studies (Table 2) investigated physical discomfort imparted by backpacks [5,7,11,13,14,35,36]. The methodical quality of these studies ranged from ‘poor’ [36] to 77% good [13]. Dockrell et al. [7] analysed musculoskeletal discomfort among primary school students and found that the prevalence of baseline musculoskeletal discomfort was high (63.4%) and that schoolbag-related discomfort was reported more frequently in the shoulders (27.3%) than in the back (15%). The dose-response assessment indicated that both statistically and practically significant increases in discomfort were observed following school bag carriage. Multiple logistic regression models indicated that psychosocial factors and a history of discomfort were predictors of schoolbag-related back discomfort, while gender (being female) and a history of discomfort were predictors of schoolbag-related shoulder discomfort. Puckree et al. [14] also found through their research that more females than males (91.9% vs. 78.5%) experienced school bag carriage pain [14].

Four of the included studies [13,14,35,36] investigated the impact of pain felt by the students from carrying their school backpacks. Two studies [13,14] focused on non-specific mechanical back pain, one study [13] looked at the regions of the body that were most affected by school backpack carriage, one study [35] analysed the influence of backpack weight on back pain and back pathologies, one study [36] analysed backpack injuries in school children and one study [11] analysed the patterns of shoulder and abdominal muscle activation during prolonged walking with loads in children. These studies found that back pain was highly prevalent in the school children, as students reported severity and chronicity of pain was high, with Siambanes et al. [13] finding that 64% of the students reported back pain, 41% felt pain when carrying their backpacks and almost all students reported relief upon taking their backpacks off their backs. Among these children, 13% rated the pain as being ‘not bad’ and 87% reported the pain to be ‘bad/very bad’. Furthermore, 16% reported that they missed days off school, gym classes and participating in sport because of the pain, while 17% of the students reported that they had seen or continued to see a doctor due to their backpack-related pain. The study by Siambanes et al. [13] found 86.9% of the school children participating in their study experienced pain in the region of the back, shoulders and neck, with most of the students relating their pain to lugging heavy schoolbags everyday [13].

In the study of Puckree et al. [14], of the 39 students, 14 reported severe pain and 20 reported moderate pain whilst carrying their backpack on both shoulders. Shoulder pain was reported most commonly, with 15% of students reporting pain in this region of the body, followed by shoulder and back pain at 7%, then neck and shoulders at 5% [14]. Rodriguez-Oviedo et al. [35] analysed the influence of backpack weight on back pain and back pathologies. 61.4% of students carried school backpacks exceeding 10% of their body weight and 18.1% exceeded 15% of their body weight; 25.9% reported having back pain for more than 15 days in the previous year, and the most frequent pathology from these reports was scoliosis (70% reported) followed by lower back pain ad contractures (10% each). Students carrying backpacks weighing in the highest quartile of loads relative to body weight had a 50% higher risk of experiencing back pain for more than 15 days than those in the lowest load quartile, with girls reporting a higher risk of back pain and an increasing risk of experiencing pain with age. It should be noted that in this study, the relative quartile loads were not provided.

Soares et al. [36] analysed backpack injuries in Indian school children, finding pressure marks (redness and swelling) over the neck and shoulders, corresponding to locations of the straps of the backpack, stooping posture while carrying the backpack, pain or stiffness in the neck, upper back and shoulders predominantly while carrying the backpack, and an absence of these symptoms during the school holidays. Soares et al. [36] also found the upper back (40%), neck (27%) and shoulders (20%) were the most prevalent body regions in which pain was reported, followed by the forearm and wrist at 7% and lower back at 6% [36]. Hong et al. [11] found that when assessing the patterns of shoulder and abdominal muscle activation during prolonged walking with loads of up to 15–20% of body weight in children, signs of muscle fatigue were found in the upper trapezius muscles after 10 min.

Five of the 21 included studies (23.8%) used questionnaires [7,13,14,15,37] asking the students for ratings of pain and body discomfort experienced while carrying their school backpack, through use of pain visual analogue scales (VAS) and ordinal scales. Five of the studies [7,13,33,37] presented results relating to backpack physical discomfort in the wearer. One study by Dockrell et al. [7] asked the student participants to answer questions linked to a body discomfort scale (BDS), and 38% of the students reported baseline discomfort (before they donned the backpack) in their shoulders and 30% reported pain in their back region while carrying the load. Students in the study were also asked questions using the VAS, with results showing a statistically significant difference between scores given before bag carriage (1.6 ± 2.3) and after bag carriage (4.7 ± 2.4) for shoulder discomfort and for back discomfort (before 2.8 ± 2.6; after 5.8 ± 2.4). This study also asked questions related to carrying their school backpack, using a strength and difficulties questionnaire (SDQ). The SDQ questionnaire is a 25-item behavior screening questionnaire, which calculates five dimensions of behavior and emotional state in 4- to 17-year-old children (e.g., emotional symptoms, conduct problems, hyperactivity, peer problems and pro-social behaviors). Parents rate each question on a grading system comprising the grades of ‘not true’, ‘somewhat true’ or ‘certainly true’, with higher scores indicating greater difficulties [7]. From these questions, the researchers found 86% of the students had a normal SDQ score, 8% were borderline, and 6% had an abnormal score [7]. The SDQ score for girls increased as their body weight increased. Another study, using a numerical pain experience scale [14], reported that 87% of students experienced bodily pain in the regions of the shoulders, with 7% feeling pain in the back and 15% feeling pain in the neck [13]. In a study by Spiteri et al. [37], the most common self-reported site of pain in students in grade 5 and 6 (8 and 9 years old) was the neck (25%), and in students in grades 7–9 (10–13 years) was the upper and lower back (44%), with a total of 8% of students in grades 5 and 6 and 17% in grades 7–9 reporting pain in multiple sites in their spines. The prevalence of self-reported back pain was higher among female students, students in non-public schools, and those in secondary schools [33].

## 4. Discussion

The aims of this literature review were to identify, critically appraise and synthesise key findings from recent studies investigating impacts of contemporary load carriage on school children, in order to inform future research and assist in the development of risk management for school backpack loads. In total, 21 publications were critically reviewed, achieving a mean ± standard deviation (SD) raw score for methodological quality of 16.86 ± 4.34 (range 5 to 21) out of 28 on the modified Downs and Black checklist [27]. Common weaknesses were identified in the included studies by the Downs and Black checklist [27]. The distributions of principal confounders in each group of subjects were typically not clearly described. External validity was often poor due to the majority of studies using controlled facilities in which to conduct assessments (for example, laboratories, classrooms and treadmills), which were not representative of the environment in which students would typically perform load carriage. For example, they often did not observe walking to and from school, walking around school grounds, walking from classroom to classroom (which may include a number of stairs), activities undertaken during recess and lunch breaks, or the associated requirement to don and doff the backpack. Internal validity also frequently scored poorly, since most of the included studies did not make an attempt to blind the participants or assessors. This was mainly due to the nature of the studies, as it would be difficult to blind participants in the studies given that participants would be aware of either their backpacks or themselves being weighed, and whether or not they were carrying loads at the time of data collection. However, given that many of the papers were rated overall as being of ‘good’ quality, the findings in general can be considered informative for the present review.

Six naturally emerging themes arose as part of the data extraction. These six themes were: (1) the participant age groups studied and contexts of study have been limited, and this warrants further research; (2) the weight of loads carried were variable but often relatively heavy; (3) the nature of loads carried are unknown (i.e., none of the students backpack contents were itemized in any of the studies); (4) the biomechanical impacts of these loads were statistically significant; (5) the physiological impacts of the loads were also significant but under-researched; (6) the pain and discomfort impacts of these loads were extensive.

### 4.1. Age Groups of Participants

Of the 21 studies included this review, only two (9%) [35,37] focused on secondary school children aged between 13–17 years. It is therefore evident that limited research has been conducted in the secondary school system (ages 13–17 years). This is important to note, given that primary school children typically carry school bags over a shorter distance and, therefore, may have a more limited exposure to load carriage [7]. A study by Mackie et al. suggests that secondary school children typically move from one classroom to another between subjects (according to their timetable) putting on and taking off their schoolbags as required and therefore carry them for longer periods of time [41].

### 4.2. Contexts of Load Carriage That Have Been Studied

Just four of the 21 studies (19%) involved students carrying their backpacks around the school on a daily basis [3,8,9,35]. Only one study (5%) [35] investigated load carriage in secondary school students carrying loads at school on a daily basis. With respect to the contexts of measurements and assessments used in the 21 studies included, in 17 studies (81%) measurements and assessments were conducted in controlled environments (e.g., laboratories, rooms and on treadmills [3,4,6,8,9,10,11,18,32,33,34,35,36,38,39] and 15 (71%) reported testing conducted with students standing still [4,5,6,7,8,10,13,14,15,32,34,35,36,37,38]. No studies involved measurements or assessments conducted in the school yard or looked at the distance travelled between classrooms or during lunch periods. With research investigating load carriage practices, noting that the contexts in which loads are carried (including the speed, terrain grade and type and distances) [5] can have a greater impact on the carrier than load weight alone, it is imperative that future research includes variation in these contextual factors as part of investigations into school child load carriage. Evaluation of the exact distances travelled whilst carrying their backpacks during a school day is needed. This may include taking into account walking to and from home, moving from classroom to classroom, movement during recess and lunch breaks and the movement after school (e.g., moving to sporting activities/venues/work/home). In addition, the types of terrain should be considered (e.g., concrete, grass or stairs), as should the speed of load carriage (e.g., walking to a classroom, running across an oval following lunch, etc.). These future research requirements were acknowledged by Lasota et al. [5] who suggested that the failure to measure the time a student spends carrying their backpack prevents the determination of the duration of load experienced, which should be considered in future studies.

Eighteen of the included studies involved students utilising their own school backpacks (double/two strapped backpack) [3,4,5,6,7,8,10,11,13,14,15,18,34,35,36,37,38,39]. Puckree et al. [14] found that the manner in which a backpack is carried (for example, carried over both shoulders or over one shoulder) significantly affected the number of children who reported pain, and so this should also be considered in future research. In that study, pain also varied with duration of load carriage, another factor that should be considered in future studies on this topic [14]. Hong et al. [9] found that no significant difference between bag types in the impacts of load carriage, with one type assessed being an athletic bag and the other a backpack, but the effects of bag type should be further investigated in future research. Typically, school backpacks lack the rigid frame of an outdoor-style backpack and include only a few pockets in the front, in addition to the main storage compartment [42]. While traditionally very simple in design, school backpacks are often made with padded shoulder straps and backs as well as additional reinforcement to support the intended load [42]. To decrease injury and improve comfort, experts recommend that children use backpacks that match the size of the child [42]. This constrains the number of items that can be carried in the backpack, and, as such may not be feasible without changes to loads needing to be carried. Backpack features from most manufactures can include elements like wide, padded shoulder straps for comfort and to distribute weight across the shoulders, padded back sections for comfort and protection, and multiple compartments for distribution of load [43]. However, these features may also add weight to the backpack itself. One area that was not discussed in the literature was the use of hip belts. A review of load carriage found that the use of hip belts affected energy costs and injury risks in tactical populations [44]. As such there may be value in investigating the use of hip belts in school bag carriage. Likewise, further research into backpack design (e.g., as mentioned above: wide, padded shoulder straps for comfort, padded back sections for comfort and protection and multiple compartments for distribution of load) is needed, particularly as materials from which the backpacks are made evolve.

Of the 21 included studies, only one paper by Pau et al. [18] addressed or mentioned alternative carrying methods (i.e., trolley bags or other kinds of bags) and this was due to these forms of bags being excluded from the study. Therefore, further research is needed to address this gap in the existing literature. In addition, several studies [44,45,46,47] have indicated that high load placement results in significantly higher levels of muscle activity than lower placement of backpacks. The differences in muscle activity are primarily due to the movements and forces arising from the angular and linear acceleration of the trunk whilst carrying load [43]. As such load placement should be considered in future research on load carriage in school children.

A study conducted by Grimmer et al. [45] also discovered that, for postural efficiency, loads should be limited to 10% of body weight and the backpacks should be worn high on the spine, considerations in agreement with load placement in the military [44]. Furthermore, the findings from this study led the authors to conclude that neither age nor gender were a significant factor when comparing postural responses to backpack loads or conditions.

### 4.3. Natures and Weights of Loads Carried

Eleven studies [5,6,8,10,11,14,18,35,37,38] weighing both the students and the backpack, reported bag loads ranging from 5 kg [37] to 7.0 kg [5]. Three studies [9,13,35] reported weighing the backpack only, with loads ranging from 0% (no load) [9] to 20% [9] of body weight. No studies were found that itemized the backpack contents (e.g., books, notebooks, stationary, toys, drinks, lunchboxes, additional uniforms, musical instruments, sporting equipment, computers etc.) nor did any study weigh an empty backpack to determine the weight of the load carriage device (i.e., backpack) itself. Lasota et al. [5] discussed the limiting impact of not investigating backpack contents, making it impossible to determine which specific elements influence the final backpack weight. In a study investigating load carriage with adult police officers, a review by Orr et al. [20] found that the average police officer carries around 7–10 kg of load (10–12% of body weight) and specialist police (like SWAT) can carry loads of around 22 kg (around 25% of body weight) as part of their occupation. While the absolute loads carried by the children in the studies included in the present review may be lighter (i.e., up to 7 kg), children have a lower total body mass than adults and as such it is evident that carried loads will be relatively heavier. As an example, Lasota et al. [5], in a study of Grade 2 children, found that the students (mean age = 8 years) were carrying up to 24% of their body weight (range 1.5–7.0 kg)—a relative load greater than those carried by general police officers and similar to those carried by elite police. This high relative load is also supported by the study conducted by Buhagiar et al. [48], who found that male children in their study (mean age = 9 years) were carrying loads of more than 20% of their body weight in the school environment.

Considering these relative loads, 14 studies in the current review reported testing backpack loads of at least 10% (range 0%/no load to 25%) of a student’s body weight [3,4,5,6,7,8,9,10,11,18,32,33,34,38]. Nearly all studies reported that a load of 10% of the student’s body weight was the appropriate maximum weight to be carried by students to limit the effects of load discomfort and injuries in agreement with the earlier recommendations by Brackley et al. [2] of a load of 10 to 15% of the child’s body weight. Conversely, Negrini et al. [21] found that around 35% of 11-year-old children in Italy carried backpacks as heavy as 30% of their body weight, stating that the average daily load of 9.3 kg carried by the school children would equate to a daily average of load of 17.6 kg for an 80 kg man. As such, determining and ensuring the optimal backpack load, relative to the student’s body weight, is a potential approach that could help mitigate the impacts of load carriage. One potential limitation of an approach of enforcing a maximum relative load, however, akin to the limitation found by militaries attempting to employ a relative loading system or protocol [49], is the requirement for a given number of items to be brought to school by the student. On this basis, if students are required to bring and carry key items (e.g., laptops, required textbooks, etc.) to school, and the weight of these items leads to a load more than the relative load recommendation, meeting a relative load recommendation may not be viable.

Harsha and Berenson [50] suggest that if students were to stop carrying a school bag they could be limiting their exposure to physical activity and even the benefits of daily resistance exercise imposed by the weight of their bag. Shultz et al. [51] go on to state that this is particularly relevant in the current situation of rising levels of obesity in children. Schools have seen a reduction in the duration and frequency of physical education classes and sport in recent times, with the battle of curriculum vs. curriculum activities. Dollman et al. [52] discuss this against a backdrop of rising youth obesity, noting that diminishing curriculum time for PE represents a serious public health issue. Dockrell [7] discusses two conflicting outcomes associated with the activity of carrying a school backpack—on the one hand it provides a form of physical exercise, and on the other it can cause physical pain or damage to a child.

### 4.4. Biomechanical Impacts of School Backpack Loads

Six of the included studies [3,8,9,33,34,39] found that backpack load carriage impacts the gait of school children, resulting in significant changes to gait parameters. Hong et al. [8] found that as loads increased to 15% of students body weight, stride length decreased, with a significant decrease in step length (*p* = 0.047), cadence (*p* = 0.057) and walking speed (*p* = 0.004) [9]. Two studies [8,10] reported findings associated with the student’s body posture, including an increase in lumbosacral angles, flattening of the thoracic kyphosis, and deepening of the cervical lordosis. Backpack loads of even 5% of body weight can significantly change trunk and lower limb angles and loads of 15% of student’s bodyweight resulted in changes in all the angles pertaining to head, neck, and lower limb, affecting overall posture [4,10]. Two of the studies found that when a backpack was worn it significantly affected foot-ground contact area and increased plantar contact pressure, especially at loads equivalent to 25% of the student’s body weight [6,18]. As such, Pal et al. [4] recommends that students should be carefully screened for possible ‘high load-high exposure time’ with a loaded backpack to reduce the risk of foot discomfort or injuries and to allow correct development of the foot structure and functionality during critical stages in a child’s physical development [6]. However, future research is needed to investigate links between load and exposure time, to quantify the level at which risk increases, and to determine whether statistically significant findings do in fact translate to clinical significance. This information can then be used to inform maximal load or maximal time dosages for backpack load carriage. Alternative carriage of the backpack by changing the positioning occasionally between anterior and posterior positions might help relieve the effects of the backpack on the spine, as identified by Chow et al. [32] who found that spinal curvature and repositioning errors were affected by backpack anterior–posterior positioning and CG levels.

### 4.5. Physiological Impacts of School Backpack Loads

The effects of backpack loads on the students’ respiratory muscle strength (inspiration and expiration) and lung function was reported in a study by Viera and Garrett [40] involving four conditions: (1) unloaded and standing still in erect stance; (2) 15% body weight load in a bilateral backpack stance; (3) 15% body weight load in a one shoulder backpack stance; and (4) 15% body weight load with a mono strap backpack, in erect stance. They found that when walking with more than 10% body weight load there was an increase in breathing rate and a decrease in trunk range of movement. The mono strap backpack, which is positioned diagonally across the body, was found to significantly lower forced vital capacity, forced expiratory volume in one second and maximal expiratory pressure. This bag design was found to restrict respiratory efforts and decrease expiratory muscle strength, so the double strap backpack is recommended as the preferred backpack option [38].

### 4.6. Physical Discomfort Caused by School Backpack Loads

Eleven of the 21 studies included provided evidence that students who were carrying backpacks recorded bodily pain, redness, swelling, fatigue and/or musculoskeletal discomfort in the upper or lower back, upper or lower trapezius, shoulders, neck or forearms, with most of the students relating their pain, fatigue and discomfort to lugging heavy schoolbags around every day—almost all students reported relief upon taking their backpacks off [5,7,11,13,14,32,33,34,35]. The prevalence of back pain was reported to be higher among female students, students in non-public schools, and those in secondary schools [33].

### 4.7. Summation of Findings

Students carried on average over 15% of their own body weight and exhibited significant biomechanical and physiological impacts of these loads as well as reporting pain, fatigue, skin redness, swelling and discomfort. Considering the limited methodological quality and variations in foci across studies, further research is needed to better elucidate: (1) the loads students carry around on a school day in their school backpacks; and; (2) the biomechanical, physiological and physical effects of load carriage on students.

From the findings in this review it is evident that there have been limited changes or improvements in student’s backpacks (in relation to load and impact) since previous reviews on this topic were conducted in 2003 [1] and 2004 [2]. The earlier review by Mackenzie et al. [1] noted that a student’s school book bag was reported to weigh more than 15% to 20% of their body weight, was associated with back pain, and when improperly used resulted in changes to the child’s posture and gait. The findings of this review were similar, with reported loads ranging up to 20% of body weight, and associations between load carriage and back pain, changes to gait and posture affirmed. Likewise, where the review by Brackley and Stevenson [2] gave maximum load recommendations for school children of 10–15% of body weight, 14 of the 21 studies in this review reported that a load of 10% of the student’s body weight was the appropriate maximum weight to be carried by students to limit the effects of load discomfort, injuries and other adverse impacts [3,4,5,6,7,8,9,10,11,18,32,33,34,38].

As a final consideration, the review by Mackenzie et al. [1] in 2003, noted that large, heavy backpacks were a relatively recent phenomenon, and discussed inadequate numbers of student lockers, less time between classes to get to lockers, larger textbooks, sports bags, musical instruments, and other objects as areas of concern regarding student loads. While the research highlighted in this review bears many similarities to that considered in earlier reviews, the contextualization of load carriage in regards to lockers, additional items being carried and travel between classrooms, and in general, was limited. As such, the load weights carried by students, backpack pain and other concerns have shown little to no improvement over the past 15 years and the recommended load of around 10% body weight is still maintained, although typically still not met. The contextualization of the carriage of these loads is also very limited. Ultimately, the concerns and unease regarding load carriage in school children remain.

### 4.8. Limitations of This Review

The literature search employed in this review was limited to two databases due to the relevance of those databases and the high numbers of papers that were identified from them. Whilst stricter search terms may have limited the overall number of initial studies identified, this approach may have meant that relevant papers were not identified in the initial search. On this basis, while limited to two databases, the search terms used were deliberately kept broad to minimize the risk of not identifying key articles.

## 5. Conclusions

Based on the methodological quality of studies included in this review and limitations in the contexts in which these studies have been conducted, it is apparent that further research into the impacts of backpack loads in real-world school settings is required using a wider range of school year levels and student ages. In particular, further research of this nature is required to assess (1) the contemporary loads students carry around on a school day in their school backpacks; (2) the impacts those loads may have on the student’s body; and (3) the biomechanical, physiological and physical effects that occur to the students as a result of carrying these loads. Despite the shortcomings of recent, available research in this area, it is nevertheless apparent that the wearing of school backpacks does have significant biomechanical, physiological and discomfort impacts on the wearer, especially with loads above 10% of the student’s body weight. Such impacts may include changes to posture (e.g., changes to spinal posture, lumbo-sacral angles, and thoracic kyphosis), gait (increases in plantar pressure during foot-ground contact and increased double support), increases in physical discomfort and muscle activity, and increases in breathing rate. Further research is required to identify and select optimal backpacks for school children, with careful consideration of the loads to be carried on a daily basis, duration of backpack load carriage and how students best carry the load to minimize the negative outcomes associated with wearing school-related backpacks.

## Figures and Tables

**Table 1:** Keywords, search terms and filters used in the literature search, by database.

Database	Date Searched	Search Terms	Filters	Results
CINAHL	30/8/2017	(Load* OR carriage OR carrying OR carried OR backpack* OR bag* OR knapsack* OR rucksack* OR pack*) AND (Child* OR adolescent* OR juvenile* OR minor* OR pubescent OR youth* OR teen*)	English Abstract available Child 6–12 years Child 13–18 years 2002–2017 publication	6522
EMBASE	30/8/2017	(Load* OR carriage OR carrying OR carried OR backpack* OR bag* OR knapsack* OR rucksack* OR pack*) AND (Child* OR adolescent* OR juvenile* OR minor* OR pubescent OR youth* OR teen*)	English Abstract available School Child 6–12 years Adolescent 13–18 years 2002–2017 publication	10,000 *

* This figure represents the maximum number of results available from the database with results ordered by ‘most relevant’.

**Table 2:** Details and findings of included studies.

Authors (Year) and Title	Participant Details	Loading Conditions	Outcome Measures Used and Context	Main Findings	Critical Appraisal Score (%)
Chow et al. (2010) Short-term effects of backpack load placement on spine deformation and responding error in school children [32].	Y6 Primary school children in Hong Kong. Mean Age 11.4 ± 0.5 years Mean Height 148.2 (standard deviation (SD) 7.7) cm 11 males and 8 females (19 Total)	Commercial backpack, double strapped. Load 15% of student’s Body Weight (BW)	Six gravitationally referenced accelerometer (ADXL311, Analog Devices (National Instruments Corporation, Austin, TX, USA) were placed on the student’s body measuring spinal curvatures of cervical, upper and lower thoracic, upper and lower lumber regions as well as pelvic tilt. Testing was conducted in a laboratory setting. Students in stationary standing position.	The objective of this study was to research the outcomes of backpack placement options on spinal deformation and repositioning error in school children. Changes to spinal curvature and repositioning error with backpack posterior and anterior positioning (*p* < 0.05) (repositioning error is associated with the backpack positioning). Less changes with backpack positioned on T12. Changing backpack from posterior to anterior helps reduce the effects on the spine.	56% = Fair
Chow et al. (2005) The effects of backpack load on the gait of normal adolescent girls [33].	Y5, Y6, Y7 and Y8 Primary and Secondary school girls in Hong Kong. Mean Age 13.4 (SD 1.1) years Mean Height 1.45–1.67 (SD 0.06) cm 22 females	Dummy designed backpack, double strapped. Load 15% load of student’s BW	Six-camera gait analysis—25 mm diameter retro-reflector markers attached to the skin surface (pelvis and lower limbs: thigh, knee, shank, ankle and foot). Walk with bare feet for 10 m, along a designed walkway in controlled environment (two force platforms mounted along the walkway). Students walking with a loaded backpack (7.5%, 10.0%, 12.5%, or 15.0% of the student’s BW) and without load.	The aim of this study was to explore the causes of carrying a variety of school backpack loads on the gait patterns of normal adolescent schoolgirls. No gross changes in gait observed Statistical analysis showed significant changes of the measured gait parameters with increased load. Increased load → decreased step length (*p* < 0.047), cadence (*p* < 0.047), walking speed (*p* < 0.004), single support time (*p* < 0.001).	52% = Fair
Dockrell et al. (2015) Schoolbag carriage and schoolbag-related musculoskeletal discomfort among primary school children [7].	Y4 and Y5, Primary school students in Ireland. Mean Age 13.4 (SD 1.1) years Mean Height 1.45–1.67 (SD 0.06) cm 55.8% (*n* = 295) males and 44.2% (*n* = 234) females (12 different schools-529 total)	Student’s own school bag loaded with school equipment and unloaded.	Students height was measured and weight under three conditions: (1) Without any bags (2) While carrying their school bags (3) While carrying their school bags and all additional items. Questionnaires were used to access information: (e.g., Body Discomfort Chart (BDC), Visual Analogue Scale (VAS), Strength and Difficulties Questionnaires (SDQ).	This study recognized schoolbag-related discomfort with the association of individual, physical and psychosocial factors. Carried bag only short distances and duration, thus limiting the exposure to load carriage as primary students leave bag in classroom all day. No physical factors were significantly associated with discomfort, but students did perceive that if a schoolbag was heavy it was also associated with an increased prevalence of back discomfort (*p* < 0.05).	74% = Good
Drzal-Grabiec et al. (2015) Effect of asymmetrical backpack load on spinal curvature in school children [4].	Y4, Y5 and Y6, Primary school students in Poland. Age 11–13 years 80 males and 82 females (126 total)	Load at 10% of student’s BW	Body posture was measured using a specialized electronic system for machine picture diagnosis called the “CQ Electronik”. Test 1. Baseline asymmetrical load with student standing up right without a backpack on. Test 2. Backpack on right shoulder (10% BW). Test 3. Backpack on left shoulder (10% BW).	This study’s aim was to assess postural parameters in the sagittal plane for an uneven/unbalanced backpack load equal to 10% of a child’s body mass. Increase in lumbosacral region angles with carriage of asymmetrical load (*p* < 0.054). Significant flattening of Thoracic Kyphosis with right shoulder load (*p* < 0.040). 10% BW load → increase in upper Thoracic spine curvature and moving head forward thus resulting in the flattening of Thoracic Kyphosis (*p* < 0.040).	70% = Good
Hong and Cheung (2003) Gait and posture responses to backpack load during level walking in children [8].	Y4 and Y5, Primary school males in Hong Kong. Age 9.43 years Height 1.34 cm 11 males total	Loads at 0%, 10%, 15% and 20% of student’s BW	Data was collected in a controlled testing environment (university gym), using a Latin Square design. Four sessions used to collect the data, students walked with set loads for each session (0%, 10%, 15% and 20% of student’s BW) for 23 laps of a basketball court (1978 m).	The aim of this study was to explore the biomechanical stresses of continuous load carriage upon children by examining the adaptations of stride and temporal parameters, and trunk posture in the school setting. Gait pattern was not altered by load but a significant increase in trunk inclination (*p* < 0.05). 20% of students BW load → significant forward lean of trunk especially as walking distance increased (*p* < 0.05).	56% = Fair
Hong and Li (2005) Influences of load and carrying methods on gait phase and ground reactions in children’s stair walking [9].	Y4 and Y5, Primary school male students in Hong Kong. Mean Age 12.21 (SD 0.98) years Mean Height 1.59.66 (SD 9.67) cm 13 males	One-strap athletic bag (across right shoulder) Double strap backpack (on both shoulders) Loads at 0%, 10%, 15% and 20% of student’s BW	Data was collected in a controlled testing environment (university gym) using a Latin Square design. An in-shoe pressure measurement system (Novel Pedar System, 99 force sensors) was used to record the temporal and kinetic data during stair walking. Students gait was assessed during 400 m flat ground walk with 20% of the student’s BW. Students then stair walked 33 steps up and down 3 consecutive times, while carrying 20% BW load.	The purpose of this study was to look at the impacts of load and backpack carrying methods on ground reaction force and gait temporal characteristics during ascent and descent stair walking in children. No significant difference between left and right foot. Athletic bag increased peak force on left foot more than right foot. Load of 15% and above of the students BW showed to induce a significant increase in stance and double support duration with the backpack on (*p* < 0.05).	74% = Good
Lasota (2014) Schoolbag weight carriage by primary school pupils [5].	Y1, Y2 and Y3 Primary school students in Poland. Age 7–9 years 54 males and 54 females (108)	Students own backpacks.	Students’ bags were weighed (by BW scales Zelmer 34Z013, SMARTFIT, Warszawa, Poland) at the start of each school day on five consecutive days (Monday–Friday). Students once at school left their bags next to their desk the entire day and did not carry them around at all.	This study researched the school-bag weight of primary school pupils (aged 7–9 years) and to identify the number of students that carried backpacks in excess of the recommended limit of 10% of their BW. No statistically significant differences in bag weights were identified (*p* < 0.647). Bag weight varied during the week.Heaviest bag: Y1—5.5 kg (*p* < 0.001), Y2—7.0 kg (*p* < 0.001), Y3—6.2 kg (*p* < 0.001).	70% = Good
Ozgul et al. (2012) Effects of unilateral backpack carriage on biomechanics of gait in adolescents: a kinematic analysis [34].	Y7 Primary school males in Turkey. Mean Age 13.2 years 20 males	Carrying backpacks at load of 15% student’s BW.	Data collection was done in a controlled environment (university laboratory). Kinematic parameters of the subject’s gait at a self-selected speed was analyzed using a six-camera motion analysis system used, with two force plates. Markers located on student’s bodies (Helen Hayes, 1991 protocol) [34]. Students were analyzed walking with no backpack vs. walking with backpack on one shoulder at a load of 15% BW.	The aim of this study was to research the biomechanical changes during walking with asymmetric backpack carrying in adolescents, focusing on the effects of asymmetrical backpack carriage on kinematic parameters of the lower body under both loaded and unloaded conditions. When carrying load, and relative to unloaded gait: ankle peak dorsal flexion increased on unloaded side and decreased on loaded side (*p* < 0.05). Mean knee varum value increased on unloaded side but decreased on loaded side (*p* < 0.05). In the sagittal plane, knee flexion increased at initial contact on loaded side relative to both the unloaded side and unloaded walking (*p* < 0.05). Hip joint maximum extension angle decreased on the loaded side compared to unloaded (*p* < 0.05). Mean hip adduction increased on loaded side and decreased on unloaded side (*p* < 0.05). Mean anterior pelvic tilt during stance increased on loaded side (*p* < 0.05). Pelvis was elevated on loaded side and depressed on unloaded side (*p* < 0.05). Both loaded and un-loaded sides were affected by asymmetrical backpack carriage. Extra load on lumbar vertebral joints and altered frontal knee biomechanics → increase in back pain and pathologies in the knee joint.	59% = Fair
Pau et al. (2011) Effects of backpack carriage on foot–ground relationships in children during upright stance [6].	Y1, Y2, Y3, Y4 and Y5, Primary school students in Italy. Mean Age 6.75 (SD 0.06)–10.82 (SD 0.07) years Mean Height 120.68 (SD 1.27)–145.12 (SD 1.27) cm 231 males and 216 females (3 different schools—447 total)	Student’s own loaded backpack.	Students first removed their shoes, then their height, body and backpack weight were all recorded. Students stood on a pressure plate with and without backpack load on. Students used their own school backpacks as per a normal school day.	This study investigated the outcome of backpack carriage in primary school children from the point of view of possible changes occurring in the foot-to-ground relationship. Foot–ground contact was significantly affected by the backpack presence (*p* < 0.01). Significant effect of the backpack only on forefoot and midfoot regions (*p* < 0.01). Load up to 10% BW increased plantar pressure in the midfoot and forefoot. Heavy load and exposure time → increase in foot discomfort.	66% = Good
Pau et al. (2015) Short-term effects of backpack carriage on plantar pressure and gait in schoolchildren [18].	Y1, Y2, Y3, Y4, Y5, Y6, Y7 and Y8, Primary and Secondary school students in Italy. Mean Age 6.8 (SD 0.3)–13.4 (SD 0.3) years Mean Height 116.8 (SD 5.0)–161.5 (SD 7.3) cm 109 males and 109 females (3 different schools—218 total)	Students own backpack mean weight 5.2 kg. Load 15% of student’s BW.	Students removed their shoes, then stature, BW, and backpack weight were all recorded. Static plantar pressure distribution measured in upright stance in quiet controlled conditions was acquired (using a pressure platform). Students were asked to standing still for 10 s then asked to walk with and without their school backpack.	The purpose of this study was (1) to evaluate the effects of backpack carriage on planter pressure quantity and issuing, and on spatio-temporal parameters of gait; (2) to examine the association between carried load and plantar pressure parameters. Spatio-temporal gait not affected by load Significantly increased contact areas in the forefoot, midfoot and rear foot (*p* < 0.001). Significant increase (up to 25%) in plantar pressure during both standing and walking on the fore foot (*p* < 0.001).	77% = Good
Puckree et al. (2004) School bag carriage and pain in school children [14].	Y7 Primary school students in South Africa. Mean Age 12.2 (SD 0.8) years 57.5% Indians, 41.0% Blacks and 1.5% Colored’s (4 different schools—195 total)	Student’s own backpack.	Students were divided into two groups: (a) Students who carried backpacks and group; and (b) Students that carried single strap backpacks. The manner in which the bag was carried was then divided into three categories: Students that carried bags over both shoulders; Students that carried bags over one shoulder; Students that carry single strap over one shoulder. Pain was assessed via a questionnaire (open and closed-ended questions). Students experiencing pain were then divided into those who’s bag mass exceed 10% of the student’s BW. The students bags were then weighted using calibrated digital bathroom scales.	This study focused on the relationship between school bag carriage and pain in school children. Examined bag type, individual characteristics, the load carried and the pain experience. Type of school bag, the manner it is carried in and the gender of the student all associated with level of ‘Shoulder pain’ (*p* < 0.001). More females experienced pain than males (*p* < 0.01).	70% = Good
Ramprasad et al. (2009) Effects of backpack weight on postural angles in preadolescent children [10].	Y7 Primary school males in India. Mean Age 12.5 (SD 0.5) years Mean Height 142.5 (SD 7.4) cm 410 males (6 different schools)	Student’s own backpack weight measured at 5%, 10%, 15%, 20% and 25% of student’s BW.	Students removed their shoes and were asked to stand on a force plate, where their weight was recorded before any measurements were taken. Students then stood on a stadiometer and their height in centimeters was recorded. Image Tool version 3.0 digitizing software (University of Texas Health Service Centre, San Antonio, TX, USA) was used for analyzing photographs.	The aim of this study was to explore the changes in postural angles under various backpack loads in preadolescent children. Craniovertebral Angles changed significantly after carrying 15% BW load (*p* < 0.05). Head and Neck angles changed after carrying 10% load BW (*p* < 0.05). Trunk and lower limb angles changed after carrying 5% BW load (*p* < 0.05). Backpack load changed all body angles and affected overall posture.	74% = Good
Rodrigues-Oviedo et al. (2012) School children’s backpacks, back pain and back pathologies [35].	Y6, Y7, Y8, Y9, Y10, Y11 and Y12 Primary and Secondary school students in Spain. Age 12–17 years Males and females (11 different schools—1403 total)	Student’s own backpack. Mean weight 7 kg.	Students were weighed with their school backpacks twice on digital scales (with a height meter). Student’s height was obtained and recorded. Questionnaire was also used to obtain information (information relating to the students lifestyle, including duration and frequency of their sport and sedentary activities). 61% carried 10% BW 18% carried 15% BW	The objective of this study was to examine the effect of backpack weight on back pain and back pathologies. Heaviest backpack resulted in an increase of back pain. Most students carried loads above 10% BW. Backpacks altered posture and gait, resulting in modifications to the head–neck angles, shoulder asymmetry and lumber lordosis. Girls had a higher risk of back pain and an increased risk with age (60.2%).	52% = Fair
Soares et al. (2012) Backpack injuries in Indian school children: risk factors and clinical presentations [36].	Y7 Primary school students in India. Mean Age 12.94 (SD 4.53) years. Mean BMI 16.70 (SD 2.81) 45% males and 55% females (22 Students)	Student’s own backpack which recorded a mean load of 5.57 kg.	Main diagnostic criteria were pressure marks (redness or swelling) over neck and shoulders, stooping shoulders, pain or stiffness in neck, upper back, shoulder. Body regions were categorized into seven regions for clinical evaluation for the collection of signs and symptoms (i.e., neck, upper back, shoulders, forearm, and wrist, lower back, thigh and wrist).	The objective of this study was to determine the risk factors and clinical presentation caused by backpacks among Indian school children. Pain results: 40% upper back, 27% neck, 20% shoulders, 7% forearm and 6% lower back. All had pressure marks over shoulders. 54.55% had myofascial pain and the rest Thoracic outlet syndrome.	22% = Poor
Spiteri et al. (2017) Schoolbags and back pain in children between 8 and 13 years: a national study [37].	Y5, Y6, Y7, Y8 and Y9 Primary and Secondary school students in Malta. Age 8–13 years 50% males and 50% females (63 primary schools and 48 secondary—134 schools = 3852 Students)	Student’s own backpack recorded median overall bag weight of 5 kg.	Students were asked questions through the use of a body chart, pain intensity (using a face pain scale-revised) frequency and consequence of back pain (questions asked: bag type, how the bag was carried, the use of lockers, participation in sport, presence of back pain, pain location). Students weight and bag weight was recorded using stadiometer scales. Students also underwent a face-to-face interview with a physiotherapist.	The purpose of this study was to assess the presence of back pain in school children, as well as its link with school bags. Over 70% had a backpack that exceeded the 10% BW level. 32% complained of back pain, but 74% said it was low intensity pain (*p* < 0.001). Self-reported pain in school children is independently linked to carrying heavy schoolbags (*p* < 0.001).	59% = Fair
Hong et al. (2008) Effect of prolonged walking with backpack loads on trunk muscle activity and fatigue in children [11].	Y1 Primary school male students in China. Age 6 years 15 male students in total.	Student’s own backpack. Loads at 0%, 10%, 15% and 20% of student’s BW.	Data was collected in a laboratory over four trials with different backpack loads for each student. Students were told to wear specific clothes for the trial (black shorts/tights and no shirt). Disposable surface electrodes were attached to the students right side of their body (upper trapezius and rectus abdominis). Students walked on a treadmill for 20 min with each of the 4 loads.	The aim of this study was to explore the effects of lengthy load carriage on muscle activity and fatigue in children when walking. Increased muscle activity was recorded at all loads (*p* < 0.05). 15% BW load significantly increased muscle activity in upper traps (*p* < 0.05). During prolonged walking, the 20% BW load was associated with the most significant muscle activity in upper and lower traps (*p* < 0.05). Fatigue in upper traps was identified within 10 min under load and in lower traps within 15 min (*p* < 0.05). No increased muscle activity found in rectus abdominus at any load or any duration of walking (*p* < 0.05).	63% = Good
Vieira (2015) Impact of backpack type on respiratory muscle strength and lung function in children [38].	Y5 Primary school students in Portugal. Mean Age 10.8 (SD 0.8) years. Mean Height 148.6 (SD 7.1) cm 12 males and 25 females (37 Students)	Student’s own backpack. Load at 15% of student’s BW.	Test done in controlled setting at the student’s school, using their own school bags. Height and weight was recorded using stadiometer and a scale). Lung function and respiratory muscle strength data was measured in the following settings: (1) Unloaded erect standing position (without the backpack). (2) Bilateral shoulder strap carried over both shoulders (with 15% of the students BW). (3) Bilateral shoulder strap carried over one shoulders (with 15% of the students BW). (4) Padded, adjustable mono shoulder strap. Inspiratory and expiratory muscle strength was tested with a digital mouth pressure meter, student exhaled slowly then inhaled with force for two seconds (repeated 3 times)	The aim of this study was to examine the effects of the backpack type on students’ lung function and strength of inspiratory and expiratory muscles in children (aged between 10 and 12). Mono strap restricted and affected lung function, decreasing expiration and muscle strength (*p* < 0.001). Double strap backpack a preferable option (*p* < 0.001). Walking with a backpack more than 10% BW increases trunk forward lean and increases breathing rate and decreases trunk Range of Movement (*p* < 0.001).	63% = Good
Cottalora et al. (2003) Influences of school bag carrying on gait kinetics [3].	Y6 Primary school students in France. Mean Age 12.2 (SD 0.5) years Mean Height 152 (SD 8.0) cm Males and females (41 Students)	Set double-strapped backpack. Load 10 kg of student’s BW	Parents asked to complete a questionnaire relation to gait patterns and movement (locomotion, physical activity and disease) and backpack carrying methods of their children. Students gait was assessed in a controlled environment (3 trials) Students were asked to walk bare-foot on an ADAL (Techmachine, Andre’zieux Bouthe’on, France) treadmill for 3 min at 3.5 km/h. Students were tested with the both the school backpacks straps on and then carrying the pack with one/single strap on.	This study determined the impact of a variety of methods of how students carry their book bags on gait kinetics (children age 11–13 years). Treadmill ergometer measured the student’s ground reaction forces of their right and left feet. Carrying a backpack on both shoulder is the preferred method. Carrying the backpack increases the students stride, stance and double stance. Vertical forces increase with the use of just one strap.	63% = Good
Siambanes et al. (2004) Influence of school backpacks on adolescent back pain [13].	Y7 and Y8 Middle school students in California USA. Mean Age 12.75 yearsMean Weight of backpack 9.33 kg 49.5% males and 50.5% females (4 schools and 3497 students in total)	Student’s own backpack	Student’s shoes were removed and they were weighed as well as their backpacks (using two calibrated digital electronic scales). The content of the backpack was not assessed. Students were then sked 22 questions in an administered questionnaire (chronicity, prevalence, severity and frequency of back pain).	This study identified the associations between school backpack weight (recorded as a percentage of the student’s overall BW), how the students wore their backpack, how long they carried the backpack, their socioeconomic status, and the prevalence, severity and chronicity of back pain. Students who walked to and from school and method of wearing the backpack were associated with level of pain. 64% reported back pain. 41% felt pain whilst carrying backpack. Girls reported a higher level of back pain.	77% = Good
Goodgold et al. (2002) Backpack use in children [15].	Y5, Y6, Y7 and Y8 primary and middle school students in Boston USA. Age 11–14 years 169 males and 176 females (345 students in total)	Student’s own backpack.	Questionnaire was used to gather information (student demographic, play and leisure activity levels, bag type and carrying methods). Students were weighed with and without their normal school backpack. Students reported they did not carry their backpacks around with them on a school, they store them in lockers.	The purpose of this study was to describe backpack use by children to assess the severity of the problem. The study examined: (1) Common backpack type used. (2) Average backpack load. (3) Backpack pain. (4) Association between history of back pain and back pack load. (5) Whether children who wear a backpack that is greater the 15% of their BW were able to identify that the load was heavy. 97% of the students do not carry the school backpack around on the school day; they use lockers. Younger students were found to carry heavier loads. Y5—19% BW. Y6—21% BW. Y7—14% BW. Y8—15% BW.	77% = Good
Connolly et al. (2008) Effects of backpack carriage on gait parameters in children [39].	Y7 Primary school students in Tennessee USA. Age 12–13 years 15 males and 17 females (32 students in total)	Student’s own backpack, 1 and 2 shoulder straps tested. Load at 15% of student’s BW	GAITRite system was used to test the students’ walking patterns (six sensor pads) measuring temporal and spatial gait parameters. Electronic walkway was used in controlled environment. Students walked under the backpack load and under no-load a total of 8 m.	This study investigated the effects of different backpack carrying methods on school-aged children. No significant difference found during loaded walk with student’s base of support, stride length and velocity when compared with the unloaded walk. Double limb support significantly increased with the loaded walk (little difference between one strap carry or two strap carry). Little change in temporo-spatial gait parameters with 15% BW load when compared with no load.	63% = Good
